# Depth Map Upsampling via Multi-Modal Generative Adversarial Network

**DOI:** 10.3390/s19071587

**Published:** 2019-04-02

**Authors:** Daniel Stanley Tan, Jun-Ming Lin, Yu-Chi Lai, Joel Ilao, Kai-Lung Hua

**Affiliations:** 1 Department of Computer Science and Information Engineering, National Taiwan University of Science and Technology, Taipei 10607, Taiwan; D10515805@mail.ntust.edu.tw (D.S.T.); M10515002@mail.ntust.edu.tw (J.-M.L.); yu-chi@mail.ntust.edu.tw (Y.-C.L.); 2 Center for Automation Research, College of Computer Studies, De La Salle University, Manila 1004, Philippines; joel.ilao@dlsu.edu.ph; 3 Center for Cyber-Physical System Innovation, National Taiwan University of Science and Technology, Taipei 10607, Taiwan

**Keywords:** depth upsampling, encoder-decoder networks, generative adversarial networks

## Abstract

Autonomous robots for smart homes and smart cities mostly require depth perception in order to interact with their environments. However, depth maps are usually captured in a lower resolution as compared to RGB color images due to the inherent limitations of the sensors. Naively increasing its resolution often leads to loss of sharpness and incorrect estimates, especially in the regions with depth discontinuities or depth boundaries. In this paper, we propose a novel Generative Adversarial Network (GAN)-based framework for depth map super-resolution that is able to preserve the smooth areas, as well as the sharp edges at the boundaries of the depth map. Our proposed model is trained on two different modalities, namely color images and depth maps. However, at test time, our model only requires the depth map in order to produce a higher resolution version. We evaluated our model both quantitatively and qualitatively, and our experiments show that our method performs better than existing state-of-the-art models.

## 1. Introduction

The standard digital camera tries to capture three-dimensional scenes and projects them onto a two-dimensional image plane. This process inevitably loses much information, particularly the depths or the distances between objects and the camera: small objects close to the camera will appear much larger, and big objects that are far away will appear much smaller than they actually are. This is why depth information is crucial in many tasks that rely on visual perception such as robot grasping, obstacle avoidance, and navigation, which are necessary tasks for smart homes and smart cities.

With the availability of affordable depth cameras such as Microsoft Kinect sensors, many systems are now being supplemented with depth range information to solve various computer vision tasks. However, the depth maps produced by these sensors have significantly lower resolution than the color images due to the intrinsic physical constraints of the sensors [1,2]. We desire accurate and high-resolution depth maps in order to perform many robotic tasks effectively, especially those involving higher risks, such as autonomous robots and self-driving cars, where small errors could lead to large costs.

A simple way of increasing the resolution of the depth map is to use the high-resolution color image. First, the depth map and its corresponding color image need to be aligned and registered since there would be a small offset coming from the side-by-side placements of the range and image sensors that needs to be corrected. Homographic warping and multi-view camera calibrations are sufficient to perform this task. Next, we can up-sample the depth maps and use the edges of the corresponding color image in order to refine the depth maps at the discontinuities where the values are likely to be uncertain. In many cases, this naive approach would work well. However, for objects with rich color textures, the edges of the textures will be treated as an object boundary, which would lead to unwanted texture-copying artifacts [3,4]. Another issue is when the object boundaries in the color image are not clear, which happens when the the object and its background have similar colors or low contrast.

In this paper, we propose a Generative Adversarial Network (GAN)-based framework that accepts multi-modal inputs, more specifically RGB color images and low-resolution depth maps, to generate higher resolution depth maps. GANs have been shown to model high frequency components well [5,6]. Since these high frequency components would correspond to the edges or object boundaries, it would be highly beneficial to incorporate them into the model. Moreover, as the discriminator in the GAN framework tries to separate real images from fake images, it would discourage the model from producing unrealistic values at the depth discontinuities as they would be considered as fake. While our model is trained on color images and depth maps, it only requires a single depth map input to perform super-resolution at test time. This is a highly desirable property since there are tasks, such as gesture recognition, action recognition, and event recognition, where the depth maps alone may be sufficient. This allows for faster models with a lesser memory footprint since we do not need to process the RGB image. There may also be applications with privacy restrictions, which would make it difficult to work with RGB images, as they are highly identifiable, unlike depth maps.

## 2. Related Work

Common approaches for depth map super-resolution can be grouped into two categories. One uses a single depth map as the input, and the other uses RGB images together with the depth maps as input. Single depth map up-sampling is desirable for applications where privacy is necessary or for applications where it is sufficient to use only depth maps. Aodha et al. [7] collected a set of training images containing large numbers of low-resolution patches and high-resolution patches. They then performed patch matching in order to synthesize the super-resolved depth map. Hornacek et al. [8] further extended this patch-based matching strategy by exploiting patch-wise self-similarity structures across depth resolutions. Li et al. [9], on the other hand, proposed to consider semantic object composition as an auxiliary regularization for assembling the high-resolution depth outputs. For these types of approaches, the collected training data have a high influence on the model’s performance.

Methods that use only single depth map inputs usually perform poorer than methods that use both RGB images and the depth maps as inputs. This is to be expected because of the added high-resolution information that they get from the RGB images. Algorithms with RGB-D inputs can be further divided into filtering-based methods and learning-based ones. Filtering-based methods are based on performing a weighted average of the neighboring pixels. The most common is the Bilateral Filter (BF) [10], which is an edge-preserving filter for image upsampling. Kopf et al. [11] proposed a Joint Bilateral Upsampling (JBU) framework that operates on both the high-resolution color image and the low-resolution depth map. They leveraged the color image to preserve the edges of the depth map. Chan et al. [12] and Li et al. [13] proposed an extended JBU method by providing a noise-aware characteristic, which can determine the effects of color information on the upsampled depth map. Yang et al. [14] proposed a Joint Bilateral Filtering (JBF) method, which is similar to JBU. Kim et al. [15] developed a JBF framework by integrating the numerical analysis of the local distribution in color images and depth maps. Liu et al. [16] also proposed an improved JBF method using geodesic distance for better-preserved depth edges. The above methods present some promising results, but color or lighting variations may produce incorrect discontinuity regions in the process of integrating color information into the upsampled depth map. Jung [17] proposed a filtering method that applies matching patterns between local color and depth patches to the filtering kernels. However, some ambiguous pattern assignments caused prediction errors in their results. Choi et al. [18] proposed another filtering method that suppresses texture-transfer and depth-bleeding artifacts. They first applied region classification to split the color image into different regions and processed each region differently. They used the Weighted Mode Filter (WMF) [19] as their main filter kernel. A problem with this approach is that depth edges at some regions are ambiguous due to color similarities. Lo et al. proposed a series of methods to improve this filtering method [4,20,21] and reported promising results.

Learning-based methods, on the other hand, rely on extracting information from a training dataset that can be generalized to different examples. Diebel and Thrun et al. [22] proposed a multi-labeling optimization problem based on Markov Random Fields (MRF), which defines a consistency term, which encourages consistency between depth values across resolutions, and a smoothness term, which encourages neighboring pixels with similar colors to have similar depth values. Revised MRF methods focusing on depth discontinuities were proposed in [3,23,24,25] to improve depth map super-resolution. Park et al. [26] improved the smoothness term by incorporating semi-local neighborhood information extracted from Non-Local Means (NLM) regularization and an edge weighting scheme, which enhances color details. Other learning-based methods like [27] phrased the depth map up-sampling task as a convex optimization problem with higher order regularization guided by anisotropic diffusion tensors extracted from high-resolution intensity images.

In this paper, we present a novel multi-modal GAN framework to perform the depth map up-sampling task. Our method combines the best of both worlds where we train on both the RGB color image and the depth maps, but only require a single depth map at testing time. We show in our experiments that this approach outperforms several state-of-the-art baselines.

## 3. Proposed Framework

**Figure 1:**
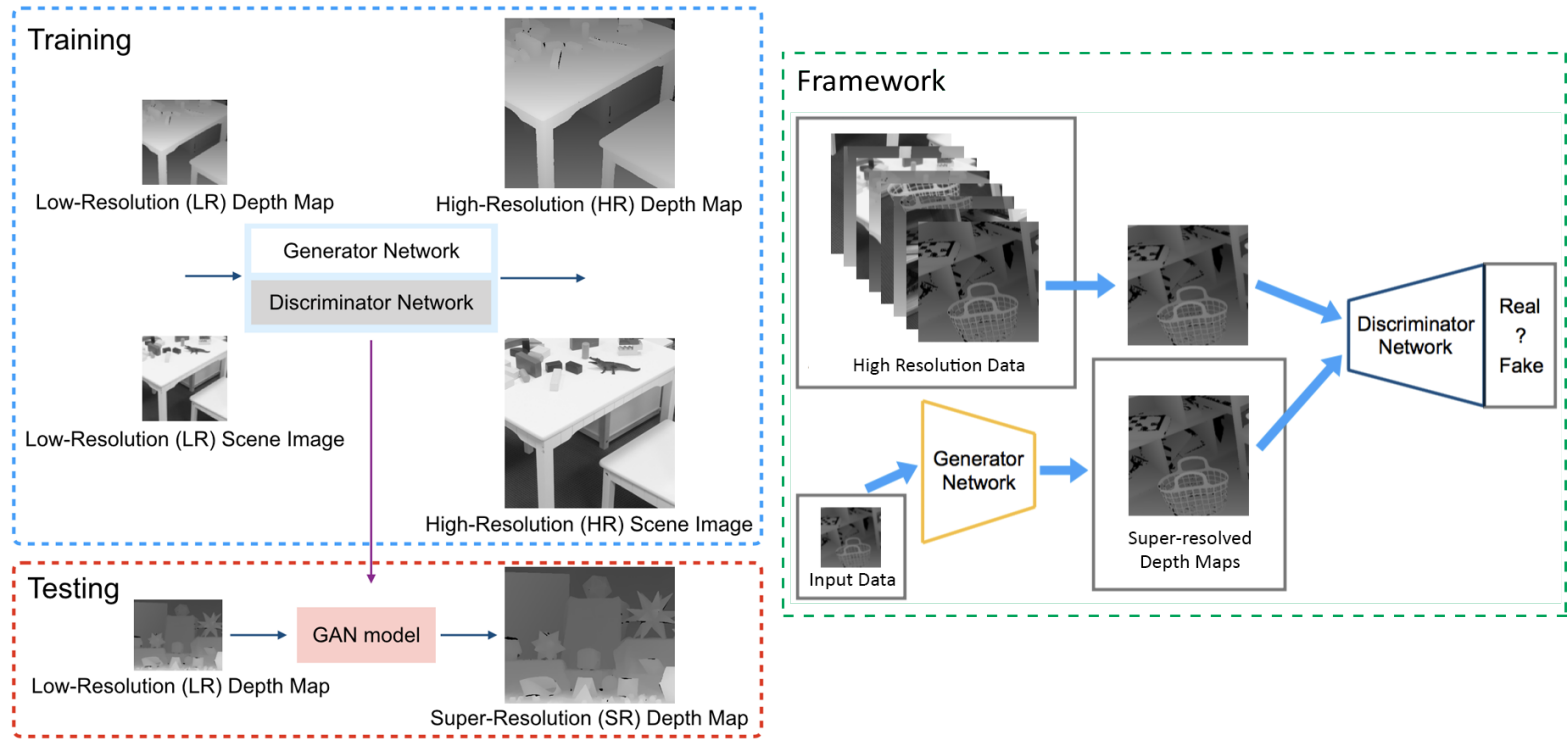
Overview of our proposed framework for depth map super-resolution.

**Figure 2:**
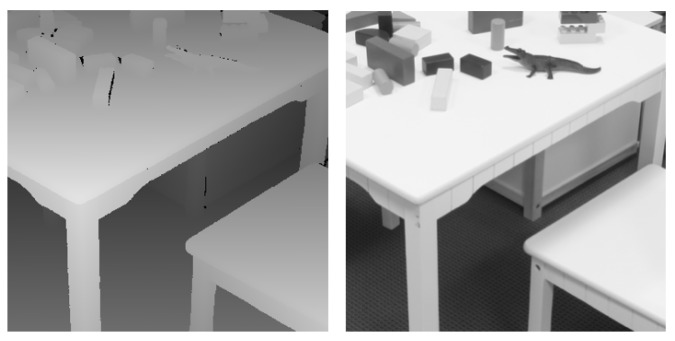
Observation of the similarities of discontinuity regions between a depth image and the corresponding scene image.

Figure 1 shows an overview of our GAN-based depth map super-resolution framework. Our generator takes inputs from two modalities: low-resolution depth maps and low-resolution scene images. Since the scene images and depth maps are structurally very similar to each other, as shown in Figure 2, we can leverage the information from the scene images to preserve the discontinuity regions of the depth maps. In order to ensure that the generator outputs valid depth maps, we train it adversarially using a discriminator that tries to distinguish whether the output of the generator is similar to real depth maps sampled from the dataset or fake depth maps synthesized by the generator.

We first formally define the problem of depth map super-resolutions in Section 3.1. Next, we discuss the details of our model starting with generative adversarial networks in Section 3.2 followed by our loss functions (Section 3.3) and network architecture (Section 3.4). Lastly, we discuss our multi-modal mini-batch scheme in Section 3.5.

### 3.1. Problem Formulation

A standard camera and a range sensor can capture a high-resolution color image $x_{i}^{HR}$ and a low-resolution depth map $x_{d}^{LR}$. Suppose that we have a dataset with the corresponding ground truth high-resolution depth maps $x_{d}^{HR}$; our goal is to learn a function $G:x_{d}^{LR}\to x_{d}^{HR}$ that can generate a high-resolution version of the low-resolution depth map. This is more commonly referred to as super-resolution. The function *G* is modeled as a convolutional neural network, which we refer to as the generator.

### 3.2. Generative Adversarial Network

The framework of Generative Adversarial Networks (GANs) introduces a discriminator *D*, which is a separate classifier, to guide the learning process of the generator *G*. This transforms the learning problem into a two-player minimax game, where the optimal solution is a Nash equilibrium.

The role of the discriminator is to learn how to tell apart real images from fake images. The generator *G*, on the other hand, is our super-resolution model, which generates high-resolution depth maps from its low-resolution input and tries to make it as realistic looking as possible in order to trick the discriminator *D* into classifying the generated depth map as real. This is represented as a min-max optimization in the form shown in Equation (1), where p(x) represents the distributions of the data and $x^{HR}$ and $x^{LR}$ are the high-resolution and low-resolution inputs, respectively. The first term accounts for the objective where we want the discriminator to classify the high-resolution inputs $x^{HR}$ coming from the dataset as real, while the second term accounts for the objective where we want to classify the super-resolved outputs of the generator ($G(x^{LR})$) from low-resolution inputs $x^{LR}$ as fake. In the formulation of these networks, the generator *G* has access to the gradients of the discriminator *D* and therefore has some form of instruction as to how to improve itself. This enables the generator to learn how to produce realistic-looking depth maps that are indistinguishable from the ground truth depth maps.

(1) $$\begin{array}{cc} \min_{G}\max_{D}L_{\mathrm{GAN}} & =E_{x^{HR}∼p\left(x\right)}\left[\log D\left(x^{HR}\right)\right]\\ & +E_{x^{LR}∼p\left(x\right)}\left[\log\left(1-D(G\left(x^{LR}\right))\right)\right] \end{array}$$

In the beginning of the training process, the fake images generated by *G* are extremely poor and are rejected by *D* with high confidence. Therefore, when performing the optimization, it is better for *G* to optimize for $\log(D\left(G\left(x^{LR}\right)\right))$ instead of $\log(1-D\left(G\left(x^{LR}\right)\right))$. Both objectives result in the same fixed point, but $\log(D\left(G\left(x^{LR}\right)\right))$ provides stronger gradients in the early stages of learning.

### 3.3. Loss Function

Our loss function is a combination of two components. The first component is a content loss or a reconstruction error, which measures how different the generated depth maps are from the ground truth depth map. It is implemented as the mean absolute error or $L_{1}$ distance between the generated and the ground truth depth map, as shown in Equation (2). As shown in previous works [5,6], this term alone will produce blurry images as the solution would turn towards the mean. However, this shows that it can capture the low frequency components well.

(2) $$L_{\mathrm{Content}}=E_{x^{HR},x^{LR}∼p\left(x\right)}\left[{∥x^{HR}-G\left(x^{LR}\right)∥}_{1}\right]$$

The second component is an adversarial loss that we have defined in the previous Section 3.2. This term encourages the outputs of *G* to reside on the manifold of the ground truth depth maps. It is also able to model the high frequency components as shown by [5,6], which makes it suitable for our problem. The final objective function is shown in Equation (3), where λ is a hyper-parameter that controls the relative importance of the two components.

(3) $$\min_{G}\max_{D}L=L_{\mathrm{Content}}+\lambda L_{\mathrm{GAN}}$$

### 3.4. Network Architecture

**Figure 3:**
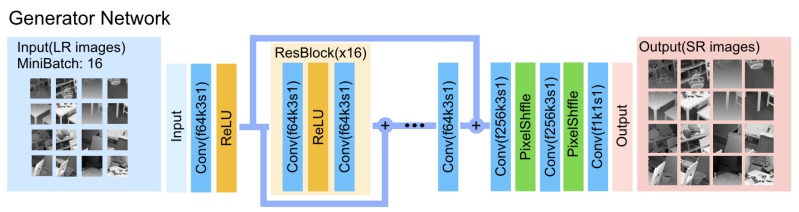
Generator network of the proposed generative adversarial network framework for depth map super-resolution with f: the number of feature maps, k: the kernel size, and s: the stride indicated for each convolutional layer.

Figure 3 details the network architecture of our generator. Inspired by Ledig et al. [28] and Lim et al. [29], we use a single convolutional layer followed by a series of sixteen residual blocks. A skip-connection is then performed, where the output of the residual blocks is combined with the output of the initial convolutional layer using an element-wise sum. The idea behind this design is that the low-resolution depth map already contains many of the pixel values for the output, and adding the skip-connection allows the network to have easier passing of information from the lower layers, which are closer to the original input, to the upper layers, which are closer to the output. We then use a two sub-pixel convolutional layer as proposed by [30], which up-samples the depth maps to four times their input resolution. We use the ReLU activation function after every convolutional layer without any batch normalization layers. We remove the batch normalization layers since it has been shown in [29] that it is detrimental to super-resolution tasks since it reduces the range flexibility by normalizing the features.

**Figure 4:**
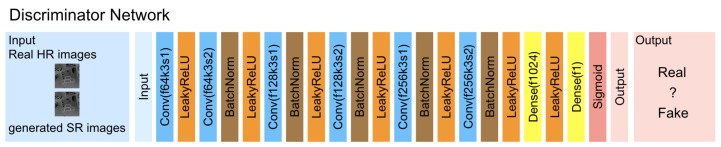
Discriminator network of the proposed generative adversarial network framework for depth map super-resolution with f: the number of feature maps, k: the kernel size, and s: the stride indicated for each convolutional layer.

As shown in Figure 4, the discriminator *D* is composed of a series of six convolutional layers where every other layer has a stride of two that downsamples the image representations by half. This is then followed by two fully-connected layers and a sigmoid activation function that outputs the probability of being real. All the layers use LeakyReLU activations, and batch normalization layers are inserted after every convolutional layer except for the first layer.

### 3.5. Multi-Modal Mini-Batch

We would like to incorporate the scene image during the learning process; however, adding an explicit branch with convolutional layers to encode and extract features from RGB scene image would require us to use the RGB image during test time. This is undesirable since our goal is to enable our super-resolution model to handle depth maps independent from the scene image at test time.

Inspired by multi-task learning and domain adaptation frameworks, we mix scene images with the depth images and perform super-resolution on both modalities during training. More specifically, we down-sample the scene image to have the same resolution as the depth map and convert it to gray scale. Now that they both have the same dimensions, we can combine them in a mini-batch and perform the training. This way, the network will be able to share what it learned from one domain (super-resolving scene images) and apply it to the other (super-resolving depth maps).

## 4. Experiments

### 4.1. Dataset

A standard dataset used widely in depth-related tasks is the NYU Depth dataset Version 2 [31]. The images and its corresponding depth maps were captured from 464 different locations coming from a variety of indoor scenes such as bathrooms, bedrooms, offices, and kitchens. We followed the same train-test experiment setup as previous studies [3,4,21]. We downsampled the ground truth depth maps from 640×480 to 160×120 (by a factor of four) in order to obtain the low-resolution depth maps to be used as the input by our model.

We also experimented on the Middlebury stereo dataset [32]. The ground truth depth maps were estimated using state-of-the-art stereo depth estimation methods that are able to handle high-resolution images. Similarly, we downsampled the depth maps by a factor of four to get our training inputs.

### 4.2. Implementation Details

We trained our model on a single NVIDIA GTX 1080 GPU with a mini-batch size of 16. During training, we randomly cropped 96×96 patches from the low-resolution depth maps, and our model up-sampled it by a factor of four producing a patch size of 384×384. Note that since our model is composed of fully-convolutional layers, it can handle arbitrary sizes at test time. We usd Adam optimizer [33] with $\beta_{1}=0.9$ and $\beta_{2}=0.999$ to train our model. Our network was trained with an initial learning rate of $1e^{-4}$. We set $\lambda =1e^{-3}$ in our loss function. We implemented our models using the TensorLayer and TensorFlow framework.

### 4.3. Performance Evaluations

Following previous works [3,4,21,32], we quantitatively evaluated our model using the percentage of Bad matching Pixels (BP%). Bad matching Pixels (BP) is defined as the percentage of pixels that have a difference larger than a pre-defined threshold from the ground truth values. This can be expressed mathematically as shown in Equation (4), where Ω refers to the set of all pixels in the depth map. To compute the percentage of bad pixels (BP%), the output depth maps have to be scaled to the same range. We used the same threshold ($\delta_{d}=1$) from previous works [3,4,21,32] to maintain a fair comparison. In addition to the whole image statistics (referenced as “all”), we also computed local statistics at non-occluded regions (“nonocc.”) and at depth discontinuity regions (“disc.”), as defined in [32,34].

(4) $$\mathrm{BP}\%=\frac{1}{\left|\Omega \right|}\sum_{\Omega }𝟙\left(\left|x^{HR}-G\left(x^{LR}\right)\right|>\delta_{d}\right)\times 100\%$$

We evaluated our model’s super-resolved depth maps at an upsampling factor of four. We compared against several state-of-the-art baselines: (1) Diebel et al. [22]; (2) JBU [11]; (3) Chan et al. [12]; (4) Lu et al. [23]; (5) Kim et al. [24]; (6) Jung [17]; (7) Park et al. [26]; (8) Ferstl et al. [27]; (9) Choi et al. [18]; (10) Lo et al. [3]; (11) Lo et al. [4]; and (12) Lo et al. [21]. We used the outputs from the respective authors’ publicly available code for the comparisons. However, there is no information on the parameter settings used in [12,22]; hence, we performed a simple grid search and reported the best performing results. Table 1 lists the performance of the various methods in terms of BP%. It can be observed that our method consistently performed better in terms of percentage of bad matching pixels.

**Figure 5:**
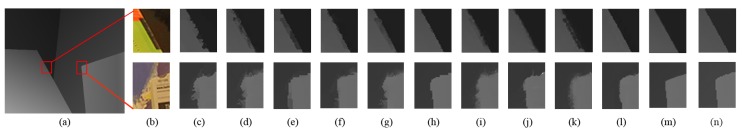
Example depth map SR results of *Venus* with two Regions Of (nterest (ROI). (**a**) Ground truth depth map, (**b**) color regions, and SR results of (**c**) Diebel et al. [22] (BP% = 1.16), (**d**) JBU [11] (0.72), (**e**) Lu et al. [23] (0.31), (**f**) Kim et al. [24] (0.30), (**g**) Jung [17] (0.59), (**h**) Lo et al. [3] (0.16), (**i**) Park et al. [26] (0.66), (**j**) Ferstl et al. [27] (0.41), (**k**) Choi et al. [18] (0.49), (**l**) Lo et al. [21] (0.16), (**m**) Our method (0.04), and (**n**) the associated ground truth.

**Figure 6:**
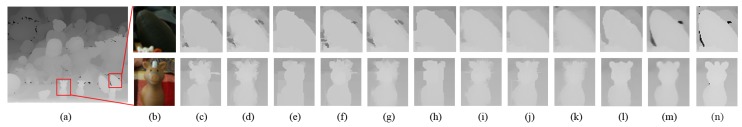
Example depth map SR results of *Dolls* with two ROI. (**a**) Ground truth depth map, (**b**) color regions, and SR results of (**c**) Diebel et al. [22] (BP% = 6.64), (**d**) JBU [11] (5.55), (**e**) Lu et al. [23] (6.69), (**f**) Kim et al. [24] (4.37), (**g**) Jung [17] (5.04), (**h**) Lo et al. [3] (3.45), (**i**) Park et al. [26] (5.07), (**j**) Ferstl et al. [27] (2.80), (**k**) Choi et al. [18] (3.17), (**l**) Lo et al. [21] (1.78), (**m**) Our method (1.74), and (**n**) the associated ground truth.

**Figure 7:**
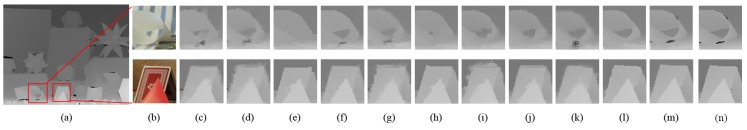
Example depth map SR results of *Moebius* with two ROI. (**a**) Ground truth depth map, (**b**) color regions, and SR results of (**c**) Diebel et al. [22] (BP% = 9.58), (**d**) JBU [11] (6.48), (**e**) Lu et al. [23] (6.36), (**f**) Kim et al. [24] (5.46), (**g**) Jung [17] (6.03), (**h**) Lo et al. [3] (4.33), (**i**) Park et al. [26] (6.49), (**j**) Ferstl et al. [27] (3.53), (**k**) Choi et al. [18] (4.97), (**l**) Lo et al. [21] (3.04), (**m**) Our method (1.53), and (**n**) the associated ground truth.

**Figure 8:**
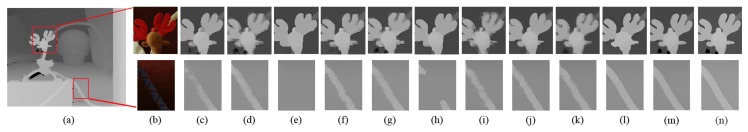
Example depth map SR results of *Reindeer* with two ROI. (**a**) Ground truth depth map, (**b**) color regions, and SR results of (**c**) Diebel et al. [22] (BP% = 3.95), (**d**) JBU [11] (3.36), (**e**) Lu et al. [23] (3.48), (**f**) Kim et al. [24] (2.14), (**g**) Jung [17] (2.91), (**h**) Lo et al. [3] (1.97), (**i**) Park et al. [26] (3.86), (**j**) Ferstl et al. [27] (1.93), (**k**) Choi et al. [18] (2.42), (**l**) Lo et al. [21] (1.42), (**m**) Our method (0.74), and (**n**) the associated ground truth.

**Figure 9:**
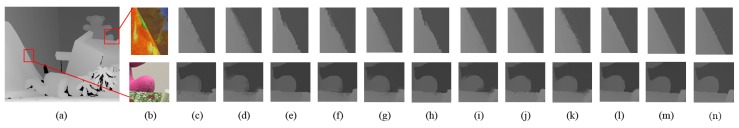
Example depth map SR results of *Teddy* with two ROI. (**a**) Ground truth depth map, (**b**) color regions, and SR results of (**c**) Diebel et al. [22] (BP% = 8.17), (**d**) JBU [11] (6.61), (**e**) Lu et al. [23] (5.60), (**f**) Kim et al. [24] (6.20), (**g**) Jung [17] (5.65), (**h**) Lo et al. [3] (3.69), (**i**) Park et al. [26] (5.24), (**j**) Ferstl et al. [27] (4.32), (**k**) Choi et al. [18] (3.88), (**l**) Lo et al. [21] (3.20), (**m**) Our method (2.76), and (**n**) the associated ground truth.

**Figure 10:**
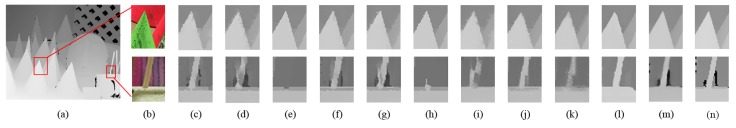
Example depth map SR results of *Cones* with two ROI. (**a**) Ground truth depth map, (**b**) color regions, and SR results of (**c**) Diebel et al. [22] (BP% = 8.10), (**d**) JBU [11] (7.54), (**e**) Lu et al. [23] (4.51), (**f**) Kim et al. [24] (5.33), (**g**) Jung [17] (5.93), (**h**) Lo et al. [3] (3.82), (**i**) Park et al. [26] (5.73), (**j**) Ferstl et al. [27] (3.44), (**k**) Choi et al. [18] (5.52), (**l**) Lo et al. [21] (3.19), (**m**) Our method (2.58), and (**n**) the associated ground truth.

**Figure 11:**
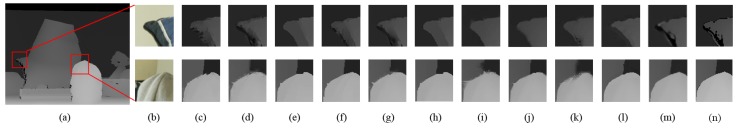
Example depth map SR results of *Midd* with two ROI. (**a**) Ground truth depth map, (**b**) color regions, and SR results of (**c**) Diebel et al. [22] (BP% = 2.96), (**d**) JBU [11] (1.95), (**e**) Lu et al. [23] (3.00), (**f**) Kim et al. [24] (2.92), (**g**) Jung [17] (2.10), (**h**) Lo et al. [3] (1.80), (**i**) Park et al. [26] (1.68), (**j**) Ferstl et al. [27] (1.40), (**k**) Choi et al. [18] (1.75), (**l**) Lo et al. [21] (0.72), (**m**) Our method (0.52), and (**n**) the associated ground truth.

**Figure 12:**
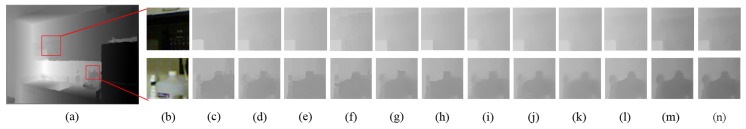
Example depth map SR results of *Kitchen1* with two ROI. (**a**) Ground truth depth map, (**b**) color regions, and SR results of (**c**) Diebel et al. [22] (BP% = 3.45), (**d**) JBU [11] (2.30), (**e**) Lu et al. [23] (2.32), (**f**) Kim et al. [24] (2.24), (**g**) Jung [17] (2.00), (**h**) Lo et al. [3] (1.45), (**i**) Park et al. [26] (2.21), (**j**) Ferstl et al. [27] (1.76), (**k**) Choi et al. [18] (1.86), (**l**) Lo et al. [21] (1.16), (**m**) Our method (0.48), and (**n**) the associated ground truth.

**Figure 13:**
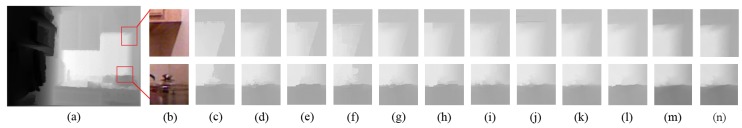
Example depth map SR results of *Kitchen2* with two ROI. (**a**) Ground truth depth map, (**b**) color regions, and SR results of (**c**) Diebel et al. [22] (BP% = 6.04), (**d**) JBU [11] (3.21), (**e**) Lu et al. [23] (3.01), (**f**) Kim et al. [24] (3.23), (**g**) Jung [17] (2.71), (**h**) Lo et al. [3] (2.96), (**i**) Park et al. [26] (3.64), (**j**) Ferstl et al. [27] (2.78), (**k**) Choi et al. [18] (2.86), (**l**) Lo et al. [21] (1.73), (**m**) Our method (0.74), and (**n**) the associated ground truth.

**Figure 14:**
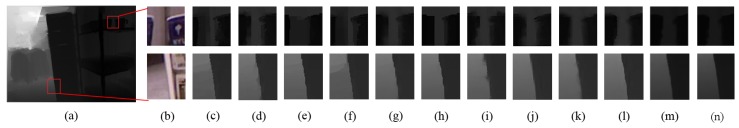
Example depth map SR results of *Store1* with two ROI. (**a**) Ground truth depth map, (**b**) color regions, and SR results of (**c**) Diebel et al. [22] (BP% = 3.09), (**d**) JBU [11] (3.60), (**e**) Lu et al. [23] (2.56), (**f**) Kim et al. [24] (2.46), (**g**) Jung [17] (2.86), (**h**) Lo et al. [3] (2.39), (**i**) Park et al. [26] (3.59), (**j**) Ferstl et al. [27] (2.24), (**k**) Choi et al. [18] (2.78), (**l**) Lo et al. [21] (2.15), (**m**) Our method (0.58), and (**n**) the associated ground truth.

We also evaluated our model using the Mean Squared Error (MSE) with respect to the ground truth depth maps. Our method was able to achieve state-of-the-art results, as shown in Table 2. MSE weights large differences more than small differences. We can observe that these large differences usually occurred at the depth discontinuity regions (“disc.”) since the local MSE values were significantly higher than the global MSE (“all”). By visually inspecting the super-resolved depth maps, we can further evaluate the performances of the various models. Figure 5, Figure 6, Figure 7, Figure 8, Figure 9, Figure 10, Figure 11, Figure 12, Figure 13 and Figure 14 show comparisons of super-resolved depth maps on several examples of patches at regions with depth discontinuity. We can see that most methods failed to capture the geometry at the edges properly. This is more noticeable when we zoom in to the patches where the straight lines were no longer straight for other methods, while our method successfully preserved these structures.

## 5. Conclusions

We proposed a multi-modal model based on the generative adversarial network for depth map super-resolution. Our method was able to preserve detailed discontinuity regions such as sharp edges, as well as respect the geometric structure of the objects. Moreover, our model only required a single depth map as an input during test time, even though it was trained with both RGB and depth maps. The results of our quantitative and qualitative experiment show that our method outperforms state-of-the-art depth map super-resolution methods, which confirms the effectiveness of our method.

## Figures and Tables

**Table 1:** Comparisons of Bad Pixel percentages (BP%) with upsampling factors of 4. For each image, the ranking of our approach is shown in parenthesis, and the best performance is shown in bold. nonocc., non-occluded; disc., discontinuity.

BP% of 4× SR	*Venus*			*Teddy*			*Cones*			*Dolls*	*Midd2*	*Moebius*	*Reindeer*	*Kitchen1*	*Kitchen2*	*Store1*
	nonocc.	all	disc.	nonocc.	all	disc.	nonocc.	all	disc.	all						
Diebel et al. [22]	0.85	1.16	3.93	7.46	8.17	18.02	6.98	8.10	19.82	6.64	2.96	9.58	3.95	3.45	6.04	3.09
JBU [11]	0.40	0.72	5.62	5.75	6.61	20.08	6.43	7.54	19.18	5.55	1.95	6.48	3.36	2.30	3.21	3.60
Chan et al. [12]	0.41	0.69	5.74	5.64	6.48	19.75	6.24	7.31	18.78	4.96	1.87	5.53	3.30	1.96	2.51	2.84
Lu et al. [23]	0.24	0.31	3.27	5.14	5.60	14.47	3.73	4.51	10.07	6.69	3.00	6.36	3.48	2.32	3.01	2.56
Kim et al. [24]	0.17	0.30	2.31	5.33	6.20	16.86	4.86	5.33	14.62	4.37	2.92	5.46	2.14	2.24	3.23	2.46
Jung [17]	0.27	0.59	3.76	4.77	5.65	15.99	4.83	5.93	14.58	5.04	2.10	6.03	2.91	2.00	2.71	2.86
Lo et al. [3]	0.12	0.16	1.67	3.35	3.69	9.59	3.15	3.82	9.43	3.45	1.80	4.33	1.97	1.45	2.96	2.39
Lo et al. [4]	0.09	0.13	1.24	3.93	4.32	12.81	3.54	3.92	10.62	2.12	0.75	3.03	1.81	1.32	1.82	2.17
Park et al. [26]	0.38	0.66	5.15	4.29	5.24	13.88	4.07	5.73	12.27	5.07	1.68	6.49	3.86	2.21	3.64	3.59
Ferstl et al. [27]	0.27	0.41	3.77	3.75	4.32	10.03	2.47	3.44	8.17	2.80	1.40	3.53	1.93	1.76	2.78	2.24
Choi et al. [18]	0.28	0.49	3.89	3.23	3.88	11.41	4.83	5.52	14.65	3.17	1.75	4.97	2.42	1.86	2.86	2.78
Lo et al. [21]	0.08	0.16	1.10	2.73	3.20	8.52	2.67	3.19	8.09	1.78	0.72	3.04	1.42	1.16	1.73	2.15
Our method	**0.04 (1)**	**0.07 (1)**	**0.57 (1)**	**2.13 (1)**	**2.76 (1)**	**6.54 (1)**	**2.16 (1)**	**2.58 (1)**	**6.54 (1)**	**1.74 (1)**	**0.52 (1)**	**1.53 (1)**	**0.74 (1)**	**0.48 (1)**	**0.74 (1)**	**0.58 (1)**

**Table 2:** Comparisons of MSE with upsampling factors of 4. For each image, the ranking of our approach is shown in the parenthesis, and the best performance is shown in bold.

MSE of 4× SR	*Venus*			*Teddy*			*Cones*			*Dolls*	*Midd2*	*Moebius*	*Reindeer*	*Kitchen1*	*Kitchen2*	*Store1*
	nonocc.	all	disc.	nonocc.	all	disc.	nonocc.	all	disc.	all						
Diebel et al. [22]	9.55	12.69	49.98	40.63	51.95	135.42	60.88	66.38	180.37	14.70	42.19	27.72	40.95	23.37	44.39	28.46
JBU [11]	2.90	5.04	36.27	61.03	72.47	224.36	80.12	84.11	241.50	17.18	25.31	23.99	51.36	7.89	20.38	10.39
Chan et al. [12]	2.77	4.79	36.41	60.78	72.17	223.87	79.79	83.71	241.06	16.35	24.32	22.58	50.62	7.35	18.61	9.37
Lu et al. [23]	6.34	25.43	39.54	16.97	20.98	**44.56**	56.16	60.36	161.66	14.02	35.73	21.88	54.25	13.76	36.51	15.59
Kim et al. [24]	2.83	3.59	19.24	40.22	51.04	141.75	61.30	63.01	182.77	13.27	31.08	14.84	29.94	11.51	38.45	15.06
Jung [17]	2.42	4.72	30.08	49.74	60.84	179.52	69.15	75.27	208.78	14.76	27.62	21.92	51.18	7.53	20.06	8.03
Lo et al. [3]	2.16	2.54	18.67	26.07	32.40	80.02	67.81	70.98	198.39	11.52	24.49	17.46	39.58	8.67	31.84	11.69
Lo et al. [4]	1.40	1.74	17.44	18.11	22.57	63.82	59.16	59.54	178.97	7.34	15.03	10.86	80.08	10.24	17.80	19.01
Park et al. [26]	2.89	4.96	35.08	49.46	57.58	111.65	49.12	83.94	141.60	11.32	20.83	16.03	64.97	9.88	17.88	14.21
Ferstl et al. [27]	2.01	3.21	29.49	39.88	50.98	109.60	42.62	58.15	97.52	9.02	19.67	10.34	44.87	5.95	18.18	12.94
Choi et al. [18]	2.38	3.44	30.65	17.34	22.56	63.24	39.17	41.94	118.48	8.72	18.06	14.03	43.75	5.55	8.42	6.64
Lo et al. [21]	1.33	2.13	16.53	**15.19**	**19.16**	51.56	41.24	42.84	124.63	**5.84**	14.00	11.25	24.01	6.57	17.72	8.95
Our method	**0.30 (1)**	**0.43 (1)**	**2.82 (1)**	17.03 (3)	22.47 (3)	62.75 (3)	**21.30 (1)**	**24.98 (1)**	**64.19 (1)**	7.97 (3)	**9.66 (1)**	**8.93 (1)**	**11.50 (1)**	**1.27 (1)**	**1.60 (1)**	**1.40 (1)**

## Abbreviations

The following abbreviations are used in this manuscript:

RGB	Red, Green, and Blue
GAN	Generative Adversarial Network
LR	Low-Resolution
HR	High-Resolution
SR	Super-Resolution
BF	Bilateral Filter
JBU	Joint Bilateral Upsampling
JBF	Joint Bilateral Filtering
WMF	Weighted Mode Filter
MRF	Markov Random Field
NLM	Non-Local Means
BP	Bad Pixel
MSE	Mean Squared Error
